# Comparison of Mitochondrial-Related Transcriptional Levels of *TFAM, NRF1 *and *MT-CO1* Genes in Single Human Oocytes at Various Stages of the Oocyte Maturation

**DOI:** 10.6091/ibj.1400.2015

**Published:** 2015-01

**Authors:** Marefat Ghaffari Novin, Mehrdad Noruzinia, Azra Allahveisi, Aboutaleb Saremi, Fateme Fadaei Fathabadi, Reza Mastery Farahani, Ali Dehghani Fard, Arash Pooladi, Ramin Mazaherinezhad Fard, Elham Yousefian

**Affiliations:** 1 *Dept. of Biology and Anatomical Sciences, Shahid Beheshti University of Medical Sciences, Velenjak, Tehran, Iran; *; 2 *Dept. of Medical Genetics, Faculty of Medical Sciences, Tarbiat Modares University, Tehran, Iran; *; 3 *Sarem Cell Research Center (SCRC), Sarem Women’s Hospital, Tehran, Iran;*; 4 *Rastegar Central Laboratory, Faculty of Veterinary Medicine, University of Tehran, Tehran, Iran*

**Keywords:** Transcription, Human oocyte, Real-time PCR

## Abstract

**Background:** The aim of the current study was to assess the mRNA levels of two mitochondria-related genes, including nuclear-encoded *NRF1* (nuclear respiratory factor 1), mitochondrial transcription factor A (*TFAM*), and mitochondrial-encoded cytochrome c oxidase subunit 1 (*MT*-*CO1*) genes in various stages of the human oocyte maturation. ** Methods: **Oocytes were obtained from nine infertile women with male factor undergoing in vitro fertilization (IVF)/*intra-cytoplasmic sperm injection* protocol. Mitochondrial-related mRNA levels were performed by single-cell TaqMan real-time PCR.** Results**: the expression level of the target genes was low at the germinal vesicle stage *(P>0.05).* Although the mRNA level of *NRF1gene* remained stable in metaphase I, the mRNA level of *TFAM* and *MT*-*CO1* increased significantly *(P<0.05).*In metaphase II, the expression level of all genes increased compared to metaphase I *(P<0.05).* **Conclusion:** The overexpression levels of *NRF1*, *TFAM*, and *MT*-*CO1 *genes are related to the oocyte maturation. Therefore, the current study could be used clinically to improve the success rate of IVF.

## INTRODUCTION

Many factors influence the fertilizability of an oocyte. Relatively, unexplained infertility is considerably seen in infertile women. In some cases, infertility occurs because oocyte fails to mature when reaches the metaphase II (MII) stage. Oocyte maturation is a well-regulated event, including nuclear and cytoplasmic maturation; both necessary for the fertilization and embryo development [1].

 Various factors affect oocyte maturation, including maturation promoting factor, gonadotropin hormones (e.g. luteinizing hormone and follicle-stimulating hormone ), insulin-like growth factor, transforming growth factor β family such as bone morphogenetic, growth differentiation factor-9, anti-mullerian hormone activins and inhibins. 

Furthermore, an extensive production and reorganization of organelles occur during the oocyte maturation. However, the mitochondrial genome must be replicated with great accuracy because mitochondria are inherited by the zygote exclusively from the oocyte [2]. Mitochondria, as the most predominant organelle in oocytes, play a critical role in this event by producing the primary supply of adenosine triphosphate through the oxidative-phosphorylation process [3]. Although altering the mitochondrial distribution (as well as their metabolic activity) known to be vital for the oocyte cytoplasmic maturation and developmental competence, little is known about the occurrence of cytoplasmic maturation during the oocyte maturation [4]. Decrease in mitochondrial activity has been reported to impair the oocyte maturation [5].

In fact, mitochondria function is associated with the mitochondrial DNA (mtDNA) [4]. A single copy of this genome exists in the mitochondria of human oocyte [6]. mtDNA number is expanded to signify-cantly larger numbers during the oocyte growth. For example, it reaches to approximately 2 × 10^5 ^in MII stage [7]. Various factors, which are dependent on the controlled coordination between the nuclear and mitochondrial genomes, have been reported to dynamically regulate the mitochondrial preservation and formation of new mitochondria [8].

 Nuclear respiratory factor1 (*NRF1*) and mitochondrial transcription factor A (*TFAM*) have been indicated to regulate mtDNA transcription and replication in various tissues. *NRF1* has been reported to be linked to promoters of *TFAM* gene and work together in mitochondrial function and biogenesis [9]. TFAM is a nuclear-encoded high-mobility group box protein, which plays its important roles by sequence binding to the heavy strand and light strand promoter sites in D-loop of human mtDNA. This promoter acts as a control region to regulate the mtDNA transcription and replication [10].

Also, it has been demonstrated that cytochrome c oxidase subunit 1 (*MT-CO1*) is the terminal component and one of the three genomic components of the mitochondrial respiratory chain [11]. Moreover, a study has shown that *MT-CO1* can be considered as an indirect indicator of activity and quantity of mtDNA [12]. It is well known that the replication initiation of the mitochondrial genome occurs at the various developmental steps in a species-dependent manner [13]. 

However, the regulation of the mitochondrial transcription in response to cell metabolic is principally unclear. A previous report has suggested a direct effect of the gene-specific transcription factors on the gene transcription in mitochondria [14]. Transcription of nuclear and mitochondrial-encoded genes during the human oocyte maturation is not well known, and no experimental data exist to describe the mitochondrial expression levels in various stages of human oocyte maturation.

Therefore, the aim of this study was to quantify the relative expression levels of nuclear and mitochondrial-encoded genes (*TFAM, NRF1, and MT-CO1*) using real-time PCR technique. The quantification assessment carried out during the various stages of human oocyte maturation from germinal vesicle to MII in a single oocyte.

## MATERIALS AND METHODS

***Sample collection. ***The oocytes of different stages were collected from nine women (20-35 years old) undergoing Intra-cytoplasmic sperm injection (ICSI) treatment due to the male factor infertility. In clinical pathology examinations, no pathological changes were seen in ovaries examined according to the following criteria: 1) medical and surgical history check, 2) clinical examinations and routine hormonal tests, 3) Rotterdam criteria use for the diseases of endocrine system, including hyper-prolactinemia, thyroid dys-function and polycystic ovary syndrome, 4) normal ovulatory period check (25–35 days), 5) body mass index records between 18.3–22.2 kg/m^2^, 6) day three based on ultrasonography, follicle-stimulating hormone check (<10 mIU/ml, estradiol < 40 pg/ml), and antral follicle count (>6), and 7) no smoking history check. Oocytes were donated by the volunteer women; from whom, more than eight oocytes were available for their own treatment. Women with female factor infertility defined as endometriosis, uterine factor, hydrosalpinx; endocrinological disorders, and history of implantation failure in previous *in vitro* fertilization (IVF)/ICSI cycles were excluded from the study. 

**Fig.1:**
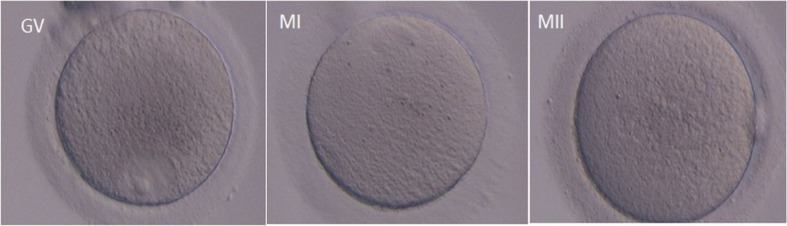
Oocytes with various maturation stages used in the study. Oocytes were selected based on their morphological criteria and classified into germinal vesicle (GV), metaphase I (MI), and metaphase II (MII).

***Treatment and donation. ***First, written approvals were signed by the patients at the Infertility and Reproductive Health Research Center, Sarem Women’s Hospital, Tehran, Iran. The study protocols were approved by the Ethical Committee of Shahid Beheshti University of Medical Sciences (Tehran, Iran). All patients were treated with gonadotropin-releasing hormone agonist in the mid-luteal phase of their previous monthly cycle (Day 21), as confirmed by serum estradiol and progesterone concentrations. Stimulation of the follicular growth was carried out by follicle stimulating hormone recombination(r-FSH) and began after sufficient down-regulation. The protocol of follicle stimulating hormone recombine-ation(r-FSH) was continued by daily injections according to the patient’s endocrine and ovarian ultrasonic responses until the observation of at least one 18-mm follicle. Then ovulation was induced using *human* *chorionic* *gonadotropin* 36 hours before oocytes collection. Vaginal puncture was conducted under the ultrasound guided IVF and ICSI protocols using transvaginal probe for collection of the cumulus-oocyte complexes. Cumulus granulose cells were mechanic-ally removed using intermittent pipetting and then enzymatically treated with hyaluro-nidase. Treatments were subsequently washed in culture media (global media). Generally, three classes of oocytes were identified based on the nuclear condition and their morphological characteristics as follow: 1) mature oocytes in MII with primary polar body (n = 8), 2) immature oocytes in metaphase I without first polar body (n = 9), and 3) Immature oocytes in germinal vesicle stage (n = 10). Figure 1 shows the stages of oocyte maturation.

***Sample processing for gene expression. ***The oocytes were processed using the Ambion Single Cell-to-CT™ Kit (Life Technologies, USA) according to the manufacturer’s manual instruction. Oocytes from various stages were transferred to RNase-free micro tubes and then processed rapidly to minimize unwanted changes. Briefly, oocytes were transferred to lysis buffer solution containing DNase I. Then, reverse transcription PCR was carried out for the samples, and cDNA was synthesized using pooled TaqMan™ Gene Expression assay for each gene prior quantitative PCR.

***Designing primers and probes. ***Primers and probes were designed using Primer Express^®^ Software v 2.0 (Table1), and all of them were then synthesized (Genfanavaran, Macrogen, Seoul, Korea).

***Gene expression analysis. ***Real-time PCR analysis was used to quantify the mRNA transcript levels of *TFAM, MT-CO1*, and *NRF1* genes in various stages of the oocyte maturation using TaqMan probes. Hypoxanthine phosphoribosyl transferase 1 gene was used as endogenous reference control. Furthermore, non-template controls were used in each PCR set. All tests were carried out in duplicates in step one real-time PCR system (Applied Biosystems, USA) using 48-well plates. Ct values were determined, and relative expression ratios were calculated using 2^-ΔΔCT^ formula.

***Statistical analysis. ***Statistical analysis was carried out using one-way analysis of variance (ANOVA) using SPSS software v16.0. Tukey's test was used to determine differences between the mean values of the gene expression levels during various stages of the oocyte maturation. *P*<0.05 was reported as significance.

## RESULTS

**Fig. 2:**
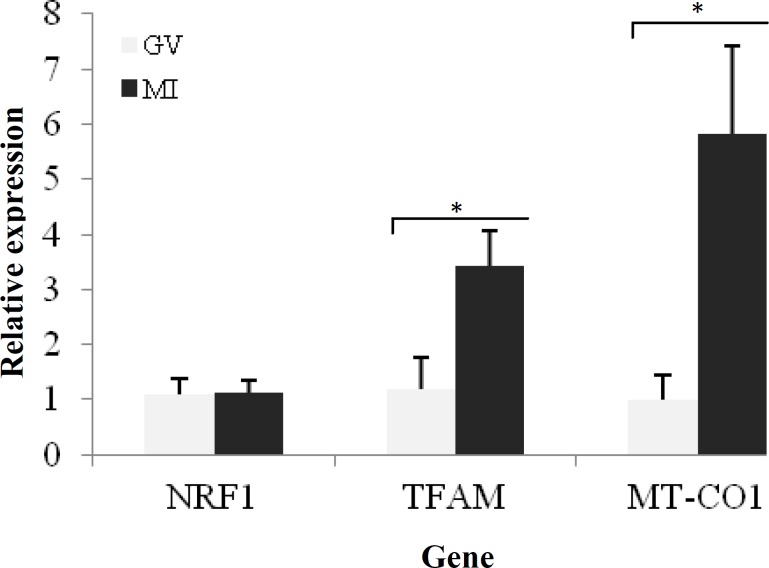
Representation of relative gene expression between germinal vesicle (GV) and metaphase I (MI) oocytes. Figure shows that two genes, *TFAM* (mitochondrial transcription factor A) and *MT*-*CO1* (mitochondrial-encoded cytochrome c oxidase subunit 1), are significantly over expressed in MI to GV oocytes (significant expression differences are shown by asterisks). *NRF1*, nuclear respiratory factor 1.

**Fig. 3:**
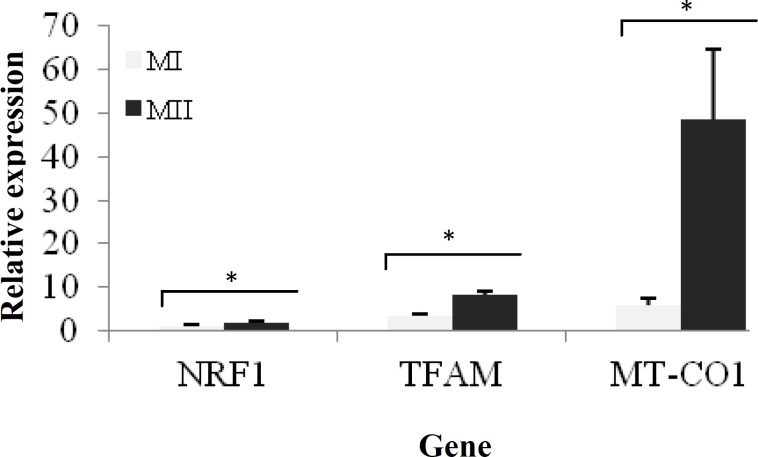
Representation of relative gene expression between metaphase I (MI) and metaphase II (MII) oocytes. Figure shows that three genes, *NRF1* (nuclear respiratory factor 1), *TFAM* (mitochondrial transcription factor A), and *MT*-*CO1* (mitochondrial-encoded cytochrome c oxidase subunit 1), were significantly over-expressed in MII compared to MI oocytes (significant over expressed genes are shown by asterisks).

**Fig. 4:**
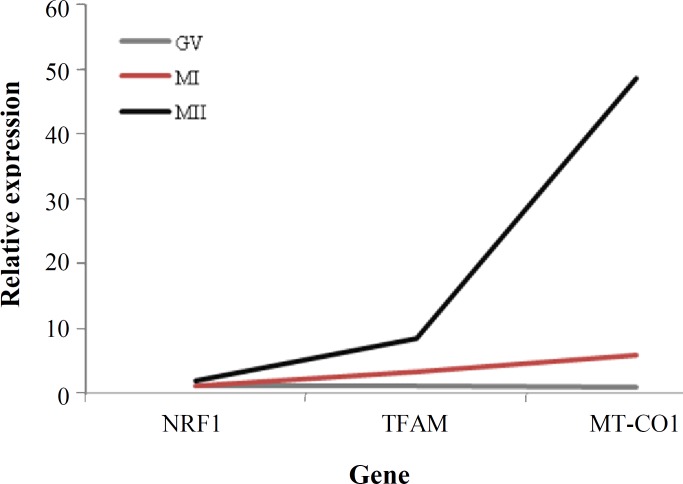
Relative quantification of the target genes at the various stages of human oocyte maturation (germinal vesicle [GV], metaphase I [MI], and metaphase II [MII]). Diagram shows trend to increase of expression level of *NRF1*(nuclear respiratory factor 1) gene in MII, compared to MI and GV; the expression levels of *TFAM* (mitochondrial transcription factor A) and *MT*-*CO1* (mitochondrial-encoded cytochrome c oxidase subunit 1) genes also shows significantly trend towards up-regulation in MII to MI and in MI compared to GV.

The *NRF1* mRNA expression was roughly stable at the metaphase I stage compared to that at the germinal vesicle stage* (P>0.05). *However, the relative levels of *TFAM* and *MT-CO1* gene expression were significantly higher in metaphase I compared to germinal vesicle stage (*P*<0.05) (Fig. 2). The results indicated a significant over-expression of *TFAM*, *NRF1*, and *MT-CO1* genes at the MII stage of the oocyte development (nearly 2.5, 1.85, and 8.34 folds, respectively) compared to those at the MI stage (Fig. 3). At the MII stage, a remarkable increase in expression level of *MT-CO1* was observed, compared to a milder increase in expression levels of *TFAM* and *NRF1* at the germinal vesicle stage (Fig. 4).

**Table1:** Primer and taqman probe sequences used in Real-time PCR

**Gene**	**Forward primer (5′–3′)**	**Reverse primer (5′–3′)**	**Probe sequence (5′–3′)**
*NRF1*	GGCACTGTCTCACTTATCCAGGTT	CAGCCACGGCAGAATAATTCA	FAM-ACCACGGTCACCGTTGCC CAA-BHQ1
*TFAM*	AAGATTCCAAGAAGCTAAGGGTGA	CAGAGTCAGACAGATTTTTCCAGTTT	FAM-CACCGCAGGAAAAGCTGA AGACTGTAAAG-BHQ1
*MT-CO1*	GAGCTGCTGTTCGGTGTCC	TGCCAGTGGTAGAGATGGTTG	FAM-CAATACCGCAACCGCATT GCCAT-BHQ1
*HPRT1*	TGGACTAATTATGGACAGGACTGAAC	GCACACAGAGGGCTACAATGTG	FAM-CTCCCATCTCCTTCATCAC ATCTCGAGC-BHQ1

NRF1, nuclear respiratory factor 1; TFAM, mitochondrial transcription factor A; MT-CO1, mitochondrial-encoded cytochrome c oxidase subunit 1; HPRT1, hypoxanthine phosphoribosyl transferase 1

## DISCUSSION

Factors that affect oocyte competency are still unknown. Nowadays, molecular methods and transcriptional analysis have become a progressively interesting approach to better understanding of oocyte development competency based on the gene expression. Many studies have been carried out to investigate the transcriptome of the competent oocytes using the gene expression analysis technique. Data extracted from this technique develop a novel knowledge to identify the mechanisms involved in this competency, which may result in the improvement of assisted reproduction techniques [15]. The current study, for the first time, determined the comparative 

expression levels of three mitochondrial-related gene transcripts at various stages of the human oocyte maturation via single-cell real-time PCR. Results showed that the expression levels of *TFAM*, *NRF1*, and *MT*-*CO1* genes were increased during the oocyte maturation. Since *NRF1* and *TFAM* genes are involved in the regulation of mtDNA copy number, it could be concluded that the relative expression levels of these genes are associated with the oocyte developmental stages. Results demonstrated that all the three target genes were expressed low in germinal vesicle-stage oocytes as compared to those in other stages.

The importance of mitochondrial function in oocyte and embryo development has been described in the previous studies [6, 13]. An investigation showed that defects in mitochondrial biogenesis or/and insufficient mitochondrial mass are associated with the failure in oocyte maturation and abnormal embryo development [16]. Several studies have indicated that mtDNA replication does not occur before blastocyst implant-ation, and functional state of mtDNA requires many factors for the gene expression, mtDNA replication, and mtDNA repair [17-19]. Therefore, embryo development depends on the existence of **adequate** *mtDNA pool* in *oocytes* to provide sufficient amounts of energy required for the various mitochondrial activities, including motility, maintenance of homeostasis, and regulation of cell survival [20]. In accordance to the current results, a study has displayed that bovine oocytes with low developmental competence and cleavage fail exhibit less expression of *TFAM*, *NRF1*, and *COX1*[21]. However, some published reports were different in various species, including pigs [22], porcine [23], mouse [24], depending on the species characteristics. In the present study, relative expression levels of TFAM and *MT*-*CO1* genes were increased in metaphase I stage oocytes compared to germinal vesicle stage. However, the relative transcription level of *NRF1* gene remained approximately stable during germinal vesicle and metaphase I stages. Nevertheless, a significant up-regulation was seen in all target genes at the MII stage compared to germinal vesicle and metaphase I stages. In MII-stage oocytes, *MT*-*CO1* showed 8.34- and 48.71-fold increase in transcript expression levels compared to metaphase I and germinal vesicle stages, respectively. This result suggests an important role for the *MT*-*CO1* gene in MII-stage oocytes. Therefore, it also could be suggested that transition from germinal vesicle to MII stage is associated with the increase in mtDNA copy numbers because increased mtDNA copies are linked to increased metabolic capacity [25]. It seems that the over expression of genes associated with RNA metabolism is important during the oocyte maturation because oocytes store a large amount of RNA to support the processes of fertilization, early embryonic development, and activation of embryonic genome.

Although the human oocyte is proposed to be transcriptionally silent at the MII stage of maturity, it seems very active in transcription and translation during the growth stages and ready to initiate transcription during the embryonic genome activation at 4- to 8-cell embryo stage [26]. Surprisingly, up-regulation of NRF1, TFAM, and *MT*-*CO1* transcripts may provide an indirect evidence for increased mitochondrial biogenesis and activation of mitochondrial respiratory activity. Mitochondrial replication and synthesis of nuclear-encoded transcripts associated with mtDNA replication are ongoing processes in growing oocytes [27]. 

The current study has demonstrated that transcription of mitochondria-related genes, which are expressed in abundant amounts, possibly affect the oocyte maturation. Furthermore, changes in the expression levels of *TFAM*, *NRF1*, and *MT*-*CO1 *genes have been shown to involve in mitochondrial transcription and replication, suggesting that most components are available for the mtDNA replication during the pre-implantation [28]. However, although many efforts have been made to reveal oocyte maturation process over the last decades, many gaps still remain. Further investigations that focus on single-cell methods may be required to better understand the unclear aspects of genes linked to mitochondria during the human oocyte maturation.

This study indicates, for the first time, that the transcript levels of mitochondrial-related (*TFAM*, *NRF1*, and *MT*-*CO1*) genes are critical factors for human oocyte maturation and subsequently oocyte quality. Additionally, results of the current study could be used clinically to improve the assisted reproductive technologies and increase the success rate of IVF.
